# Length of hospital stay after delivery among Danish women with congenital heart disease: a register-based cohort study

**DOI:** 10.1186/s12884-021-04286-3

**Published:** 2021-12-07

**Authors:** Anne Marie Kirkegaard, Maria Breckling, Dorte Guldbrand Nielsen, Janne S. Tolstrup, Søren Paaske Johnsen, Annette Kjær Ersbøll, Stine Kloster

**Affiliations:** 1 National Institute of Public Health, University of Southern Denmark, Studiestræde 6, 1455 Copenhagen K, Denmark; 2 Department of Clinical Medicine, Aarhus University, Aarhus, Denmark; 3 Department of Cardiology, Aarhus University Hospital, Aarhus, Denmark; 4 Danish Center for Clinical Health Services Research, Department of Clinical Medicine, Aalborg University, Aalborg, Denmark

**Keywords:** Congenital heart disease, Pregnancy, Length of hospital stay

## Abstract

**Background:**

The literature about the impact of congenital heart disease (CHD) on the length of hospital stay after delivery is limited, and nonexisting in a country with free and equal access to healthcare. We aimed to examine the hypothesis that Danish women with CHD have a longer hospital stay after delivery compared to women without CHD. Secondarily, we aimed to examine the hypothesis that cesarean section modifies the association.

**Methods:**

The study was a national cohort study using Danish nationwide registers in 1997–2014. Maternal CHD was categorized as simple, moderate, or complex CHD. The comparison group consisted of women without CHD. Outcome of interest was length of hospital stay after delivery registered in complete days. Mode of delivery was categorized as cesarean section or vaginal delivery. Data was analyzed using a generalized linear model with a Poisson distribution.

**Results:**

We included 939,678 births among 551,119 women. Women without CHD were on average admitted to the hospital for 3.6 (SD 3.7) days, whereas women with simple, moderate, and complex CHD were admitted for 3.9 (SD 4.4), 4.0 (SD 3.8) and 5.1 (SD 6.7) days, respectively. The adjusted length of hospital stay after delivery was 12% (relative ratio (RR) = 1.12, 95% confidence interval (CI) 1.07–1.18), 14% (RR = 1.14, 95% CI: 1.07–1.21), and 45% (RR = 1.45, 95% CI: 1.24–1.70) longer among women with simple, moderate, and complex CHD, respectively, compared to women without CHD. The association between maternal CHD and length of hospital stay was not modified by mode of delivery (*p*-value of interaction = 0.62). Women who gave birth by cesarean section were on average admitted to the hospital for 2.7 days longer compared to women with vaginal delivery.

**Conclusion:**

The hospital stay after delivery was significantly longer among women with CHD as compared to women without CHD. Further, higher complexity of CHD was associated with longer length of stay. Cesarean section did not modify the association.

**Supplementary Information:**

The online version contains supplementary material available at 10.1186/s12884-021-04286-3.

## Background

Globally, there has been an increase in the number of adults living with congenital heart disease (CHD) [1–3], due to improvement in both diagnosing and medical care. This results in more women with CHD reaching the childbearing age, and both the number and proportion of women with CHD giving birth is on the rise [4–7].

Most women with CHD will be able to complete a pregnancy, despite a larger proportion of women with CHD experiencing more cardiac, obstetric, and neonatal complications compared to women without CHD [7–11]. Several American studies have also shown an association between CHD and length of hospital stay after delivery [6, 7, 12–16] which might reflect a more complicated delivery [12], a greater proportion of deliveries by cesarean section [5, 7, 11, 12, 17, 18] and more preterm births [7–9, 11, 17] that result in hospitalization, or healthcare workers’ precautions for early discharge of women with known CHD. However, in a setting with user paid healthcare the length of hospital stay might also be influenced by insurance status and socioeconomic position [19, 20].

The length of hospital stay after delivery among women with CHD has not been investigated in a healthcare system with free and equal access as e.g. the Danish healthcare system. This information is important for healthcare workers and healthcare authorities in order to have an unbiased estimate, when planning resources directed to the growing population of women with CHD surviving into the childbearing age. Furthermore, the knowledge can help elucidate whether the length of stay differs in a public healthcare system compared to others, as one may wonder if public financed systems are more cautious to discharge patients because the patients do not pay directly. Therefore, our primary aim was to examine the hypothesis that women with CHD have a longer hospital stay after delivery compared to women without CHD in a healthcare system with free and equal access. The secondary aim was to examine the hypothesis that cesarean section modifies the association between CHD and length of stay, i.e., the association between CHD and the length of hospital stay differs depending on whether the birth is a vaginal delivery or a cesarean section.

## Methods

We performed a nationwide cohort study with data from the Danish Medical Birth Register [21, 22] and the Danish National Patient Register [23, 24]. In Denmark, all citizens are assigned a unique personal identification number, which enables individual-level linkage of national registers [25, 26].

### Study population

The study population consisted of births between 1997 and 2014, registered in the Danish Medical Birth Register. The register holds information on all live and stillbirths registered in Denmark, including information of both mother and child related to the pregnancy and delivery [21, 22]. Only births of women born in Denmark were included to secure equal opportunity for CHD diagnosing among women. We included all singleton births (*n* = 952,882).

### Maternal congenital heart disease

Information about maternal CHD was obtained from the Danish National Patient Register, which is a population-based administrative register holding information on all hospital admissions since 1977 and all outpatients contacts since 1995 [23, 24]. All women with a diagnosis of CHD (International Classification of Diseases and Health Related problems, Tenth Revision (ICD-10): Q20-Q26, International Classification of Diseases, Eighth Revision (ICD-8): 746–747) between 1977 and 2015 were included except ICD-10: Q26.5-Q26.6 and ICD-8: 746.7 and 747.5–747.9, which are not specific for CHDs. Additionally, we excluded invalid diagnoses of CHD or inaccurate coding in the Danish National Patient Register; for example by excluding diagnoses of patent ductus arteriosus if diagnosed before the age of 2 months without an associated operation code or by excluding diagnoses of congenital stenosis of aortic valve if diagnosed at ages > 40 years, as has been done in previous publications [27]. Based on available diagnoses women were categorized as having no CHD, simple, moderate or complex CHD as described elsewhere [8].

### Length of hospital stay

Outcome of interest was length of hospital stay after delivery. Information was obtained from the Danish Medical Birth Register where length of hospital stay was reported based on date of admission and discharge in the Danish National Patient Register and reported in complete days such that the shortest possible length of stay was 1 day [21, 22] . To minimize the influence of outliers and unreliable registrations of days we truncated length of hospital stay at 50 days.

### Covariates

Maternal ethnicity, age, parity, educational level, and year of delivery were identified as confounders a priori using a Directed Acyclic Graph (DAG) (Supplementary Fig. S1) [28]. The DAG was created using the free software package DAGitty [29].

Information about ethnicity was obtained from the Danish Civil Registration System [26] and grouped into emigrants or descendants with Western and Non-Western origin. Information about maternal age, year of delivery, and parity was obtained from the Danish Medical Birth Register [21, 22]. Age was categorized into four categories: < 25, 25–29, 30–34, and ≥ 35 years. Year of delivery was grouped into year-bands of 5 years, except the last interval which contained 3 years. Information about parity was grouped into nulli-, primi-, and multiparous; corresponding to never given birth before the present pregnancy, given birth once before and more than once before. In case a woman was noted as e.g. nulliparous and was registered with more than one birth during the study period, parity was corrected based on the available number of births in the study period as described elsewhere [8].

Information on mode of delivery was obtained from the Danish Medical Birth register and categorized as having a vaginal delivery or a caesarean section.

Information about the highest level of completed education registered the 1st of October the year preceding each birth was obtained by linkage to the Danish Education Register [30]. Level of education was classified according to the International Standard Classification of Education System (ISCED) [31] and categorized into three groups; low (pre-primary, primary and lower secondary; ISCED level 1–2), medium (upper secondary and postsecondary; ISCED level 3–4), and high education (tertiary education; ISCED level 5–8).

### Statistical analyses

Descriptive analysis of the study population was performed by means or frequencies (number and proportion) of births by maternal CHD. Furthermore, the timewise development in median number of days of admission after delivery were illustrated for nulli- and multiparous women according to their CHD status.

The association between maternal CHD and length of hospital stay after delivery was examined using a generalized linear model with a Poisson distribution. Women without CHD constituted the reference group. The relative ratio (RR) and 95% confidence interval (CI) of length of hospital stay after delivery was estimated for simple, moderate, and complex CHD, respectively, as compared to length of hospital stay after delivery among women with no CHD. An unadjusted model and an adjusted model were used. Confounders included in the adjusted model were maternal age, parity, ethnicity, educational level, and year of delivery. The overall *p*-value of the effect of maternal CHD was calculated using a likelihood ratio test in the unadjusted and adjusted models. Some women had more than one birth during the study period in 1997–2014. To account for the hierarchical data structure with births nested within women, a cluster-robust standard error estimator was used.

To examine if cesarean section modified the association between maternal CHD and length of hospital stay after delivery the interaction between maternal CHD and mode of delivery was included as well as the main effect of the two variables. Wald’s test was used to test the significance of the interaction term.

### Sensitivity analysis

Four sensitivity analyses were performed. In the main analysis, the length of hospital stay was truncated at 50 days to minimize the influence of outliers. In the first two sensitivity analyses, the truncation of length of hospital stay was modified to 30 and 100 days, respectively. In the third sensitivity analysis, we excluded all women with a hospital stay above 50 days. In the fourth sensitivity analysis, we used a multilevel model (i.e., a generalized linear mixed model) with two levels (women and births nested within women).

### Ethics

In Denmark, no ethical approval or written informed consent are required for register-based studies [32, 33].

## Results

### Participants

**Fig. 1:**
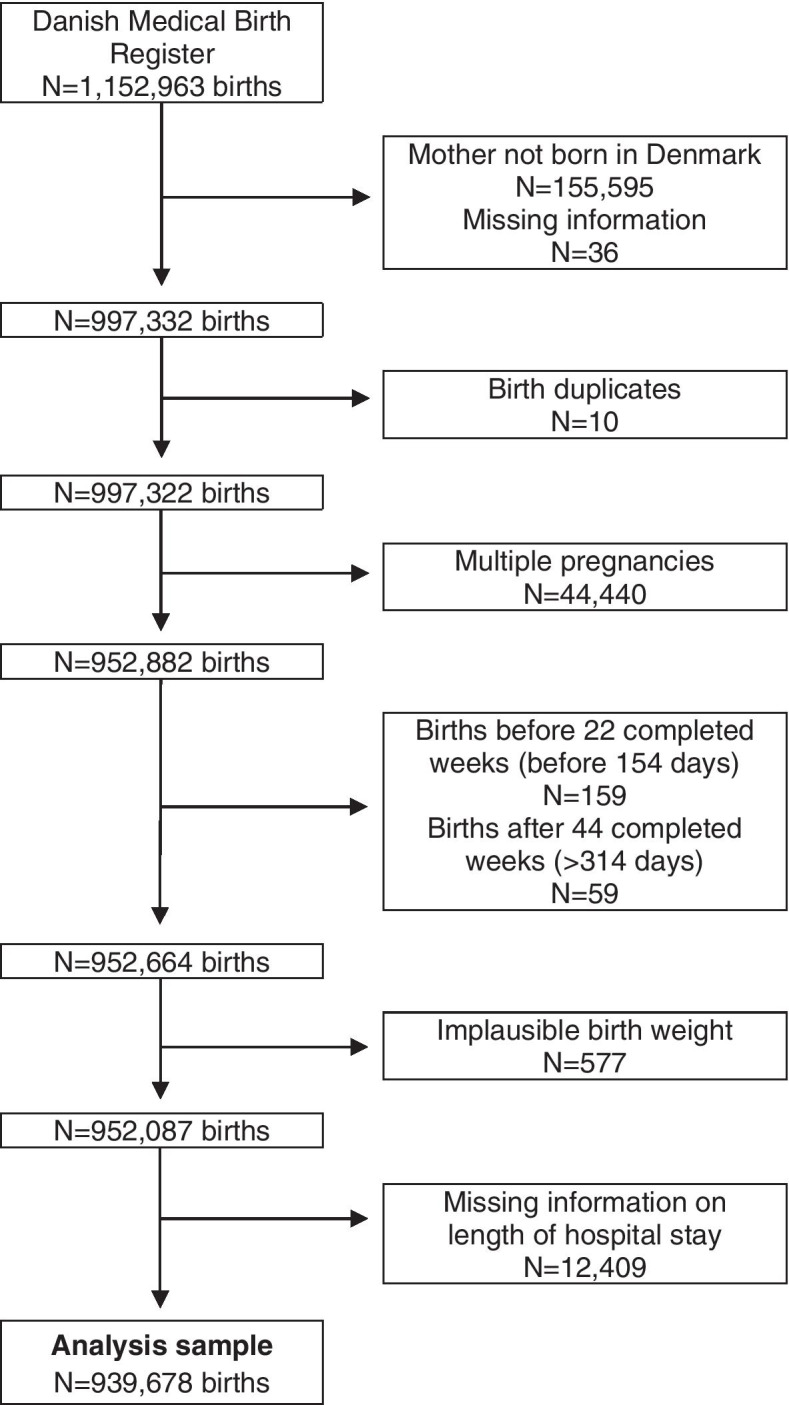
Flow diagram of data from the Danish Medical Birth Register

The population consisted of 952,882 births after exclusion of births with multiple pregnancies or missing information on maternal ethnicity. Furthermore, births completed before week 22 and after week 44 of completed gestation were excluded (*n* = 218). Also, implausible birthweights (*n* = 577) and missing information on length of hospital stay (*n* = 12,409) were excluded. Therefore, the final population consisted of 939,678 births among 551,119 women. The selection of the study population is illustrated in Fig. 1.

Baseline characteristics by CHD status are shown in Table 1. Overall, 15.7–20.0% of women with CHD were < 25 years of age at birth, 45.1–48.6% were nulliparous and 22.6–27.5% had a low education compared to 12.6, 43.9 and 19.1% of women without CHD, respectively. Additionally, a greater proportion of women with CHD had a cesarean section as compared to women without CHD (29.2% (for women with complex CHD) versus 18.5%).

**Table 1:** Baseline characteristics by congenital disease status of 939,678 births in 551,﻿119 women. Denmark, 1997–2014

	Maternal congenital heart disease							
	No		Simple		Moderate		Complex	
	n^a^	(%)	n^a^	(%)	n^a^	(%)	n^a^	(%)
**Maternal age (years)**	935,959		2242		1093		384	
< 25		12.6		20.0		15.7		19.3
25–29		33.8		32.9		36.2		35.1
30–34		36.1		33.4		34.1		34.1
≥35		17.5		13.7		14.0		11.5
**Parity**	922,147		2210		1075		381	
Nulli		43.9		48.5		48.6		45.1
Primi		38.7		36.3		36.8		35.2
Multi		17.4		15.2		14.6		19.7
**Maternal ethnicity**	935,959		2242		1093		384	
Western		98.7		97.8		98.9		96.4
Non-western		1.3		2.2		1.1		3.6
**Maternal educational level**	923,832		2210		1078		375	
Low		19.1		27.1		22.6		27.5
Medium		43.8		40.5		41.6		46.4
High		37.1		32.4		35.8		26.1
**Year of delivery (groups)**	935,959		2242		1093		384	
1997–2001		29.6		22.3		25.3		23.2
2002–2006		28.5		25.3		26.3		27.9
2007–2011		27.5		32.3		30.9		33.8
2012–2014		14.4		20.1		17.5		16.1
**Mode of delivery**	935,959		2242		1093		384	
Cesarean section		18.5		24.3		23.1		29.2

^a^Number of births

### Length of hospital stay

**Fig. 2:**
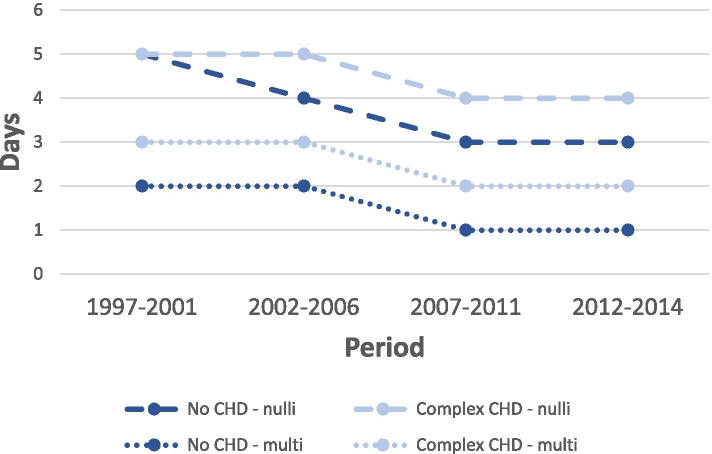
Median number of days of admission after delivery among women with complex CHD and without CHD for nulliparous women and multiparous women

The length of hospital stay after delivery was significantly longer among women with CHD than among women without CHD. Further, the length of stay was longer among women with higher severity of the CHD with average hospital stays of 3.6 (SD 3.7), 3.9 (SD 4.4), 4.0 (SD 3.8) and 5.1 (SD 6.7) days for women without CHD, simple, moderate and complex CHD, respectively (Table 2). During the study period the median number of days of admission has declined regardless of CHD status and parity with nulliparous women with complex CHD having the longest stay. The decline was similar between women without CHD and complex CHD (Fig. 2).

**Table 2:** Association between congenital heart disease and length of hospital stay after delivery given by relative ratio (RR) and 95% confidence interval (95% CI). Denmark, 1997–2014

		Number of days of admission after delivery		Unadjusted			Adjusted^b^		
	n^a^	Mean (SD)	Median (IQR)	RR	95% CI	*p*-value	RR	95% CI	*p*-value
**Maternal congenital heart disease**						< 0.001			< 0.001
No	935,959	3.6 (3.7)	3 (1–4)	1 (ref)	–		1 (ref)	–	
Simple	2242	3.9 (4.4)	3 (2–5)	1.10	1.05–1.16		1.12	1.07–1.18	
Moderate	1093	4.0 (3.8)	3 (2–5)	1.13	1.06–1.20		1.14	1.07–1.21	
Complex	384	5.1 (6.7)	4 (2–5)	1.42	1.22–1.66		1.45	1.24–1.70	

^a^Number of births in the unadjusted analysis

^b^Adjusted for maternal age, year of delivery, parity, ethnicity, and maternal educational level

**Fig. 3:**
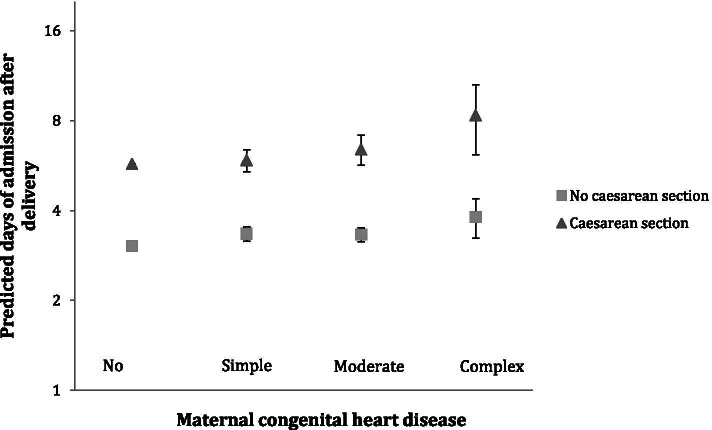
Predicted days of admission after delivery by maternal congenital heart disease status and caesarean section. P-value for interaction is 0.62. Adjusted for maternal age, parity, ethnicity, educational level and year of delivery﻿

The length of hospital stay after delivery was extended with 45% (adjusted RR = 1.45, 95% CI: 1.24–1.70), 14% (RR = 1.14, 95% CI: 1.07–1.21) and 12% (RR = 1.12, 95% CI: 1.07–1.18) for women with complex, moderate, and simple CHD, respectively, compared to women without CHD (Table 2). There was no difference in length of hospital stay among women with moderate and simple CHD (*p* = 0.70). The association between maternal CHD and length of hospital stay was similar in strata of mode of delivery (*p*-value of interaction = 0.62, Fig. 3). Women who had a cesarean section had on average 2.7 days longer hospital stay after delivery compared to women who did not give birth by cesarean section (Fig. 3).

### Sensitivity analyses

The sensitivity analyses with length of hospital stay truncated at 30 days (Supplementary Table S1) and 100 days (Supplementary Table S2) after delivery gave almost the same results as the main analysis (truncated at 50 days; Table 2). Also, exclusion of women with hospital stays above 50 days gave similar results (Supplementary Table S3).

Additionally, modeling the association using a multilevel analysis did not change the results compared to the main analysis (Supplementary Table S4).

## Discussion

This nationwide register-based cohort study found that the length of hospital stay after delivery was significantly longer among women with CHD as compared to women without CHD. The hospital stay was 45% longer among women with complex CHD, 14 and 12% longer among women with moderate and simple CHD compared with women without CHD. Cesarean section did not modify the association between maternal CHD and length of hospital stay after delivery.

Our findings are supported by similar American studies, where length of maternal hospitalization on average was longer among women with CHD compared to women without CHD [6, 7, 12–15]. One study reported that 23.8% of women with CHD stayed > 3 days after delivery as compared to 13.4% of women without CHD [6]. Likewise, Warrick et al. [15] reported that almost half of all deliveries complicated by maternal CHD resulted in a prolonged hospital stay after both vaginal delivery and cesarean section. Additionally, Hayward et al. [14] found that 11.1% of women with complex CHD and 6.0% of women with non-complex CHD had a hospital stay more than 7 days after delivery as compared to 1.3% among women without CHD. Furthermore, a recent American study reported a mean hospital length of 2.3 days among women with CHD as compared to 1.8 days among women without CHD [12]. Also, they reported a longer hospital stay among women with more complex CHD as compared to women with simple CHD; 2.6 days versus 2.2 days. These are shorter stays compared to the length of stay in the current study. However, free and universal healthcare, as well as national guidelines most likely affect the current practice and length of hospital stay. Nevertheless, despite differences in the absolute length of stay, the pattern between women with and without CHD was similar to previous studies.

A longer admission after delivery among women with CHD can be a consequence of several factors that are more common among women with CHD than among women without CHD, e.g. a higher proportion of preterm birth [8, 9, 11, 14, 17], obstetric complications or cardiac complications during pregnancy and delivery [7, 10, 17, 34, 35]. It could involve a pathway from CHD to obstetric complications during pregnancy and delivery leading to a higher frequency of cesarean section and longer hospital stay. In line with the exiting literature, we found a higher proportion of women with CHD having cesarean section as compared to women without CHD [5, 7, 11, 12, 14, 15, 17, 18, 36]. Further, we found that the length of hospitalization was longer after a cesarean section among both women with and without CHD. However, in the present study we show that the length of hospital stay is not prolonged additionally after a cesarean section among women with CHD as compared to women without CHD. This finding indicates that cesarean sections do not affect women with CHD more severely than women without CHD; at least not to an extent that is reflected in longer hospitalization. However, a longer hospital stay could also be due to caution among clinical staff. This need to be addressed in future research in order to understand if longer stay was a positive or negative outcome and further if it should or could be prevented.

There is an international trend to shorten the postpartum hospital stay among healthy women [37]. However, both early discharge and longer hospital stay among healthy women has been speculated to be associated with adverse outcomes as e.g. postpartum depression, readmission and neonatal mortality rate [37, 38]. Among women with CHD other factors might need consideration, e.g., Hayward et al. [14] showed that women with CHD more often were readmitted to the hospital within 30 days, 1 year and 7 years after delivery-related discharge as compared to women without CHD. Therefore, the association between length of hospital stay and subsequent adverse maternal and neonatal outcomes needs to be investigated among women with CHD. Analyzing the number of readmissions was beyond the scope of this study, however, it may indicate how a prolonged hospital stay among women with CHD is associated with readmission rate after delivery in a Danish setting.

### Strengths and limitations

The main strength of the present study was the inclusion of all women in Denmark diagnosed with CHD, minimizing the risk of selection bias compared to inclusion from specialized clinics. Further, it was possible to include women with simple CHD who often give birth in non-specialized hospitals.

A further strength of our study is the validation of CHD diagnoses. A recent American study has shown that CHD diagnoses from administrative databases are associated with inaccuracy [39]. In Denmark, CHD diagnoses are in general associated with a high positive predictive value [40, 41]. However, to increase the validity further we used an algorithm previously described (see appendix in [27]) to exclude invalid diagnoses of CHD and inaccurate coding.

Further, we find our results to be robust since similar results were found in the sensitivity analyses where the length of admission were truncated at different lengths. For a limited number of births (1039 births, 0.11%) the hospital stay was > 50 days and considered incorrect, most likely due to an error in the date of discharge. Therefore, number of days of admission was truncated to 50 days. However, changing the truncation to 30 days and 100 days, respectively, or excluding these observations resulted in similar results and this bias is therefore considered to be minor.

The main limitation is that the clinical data, such as blood pressure and electrocardiogram, were not accessible and, therefore, we were unable to differentiate between severity within a given CHD diagnosis when categorizing CHD into simple, moderate, and complex.

## Conclusion

This nationwide register-based cohort study found that the hospital stay after delivery was significantly longer among women with CHD as compared to women without CHD. Further, length of stay increased with increasing CHD complexity. Cesarean section did not modify the association between maternal CHD and length of hospital stay after birth, indicating that a cesarean section does not affect a woman with CHD more severely than a woman without CHD; at least not to an extent that was reflected in a longer hospitalization. The results can help to highlight the need for future expenses and amount of hospital capacity in relation to prolonged hospital stays after delivery for the growing population of women with CHD. Furthermore, our results, together with the existing literature, indicate that the pattern in hospitalization is similar across private and public healthcare systems.

## Supplementary Information

**Additional file 1 **: **Figure S1**. Simplified Directed Acyclic Graph (DAG) highlighting variables of importance in the analysis of the association between maternal congenital heart disease and length of hospital stay. **Table S1**. Association between congenital heart disease and length of hospital stay after delivery given by relative ratio (RR) and 95% confidence interval (95% CI). Length of hospital stay is truncated at 30 days. **Table S2**. Association between congenital heart disease and length of hospital stay after delivery given by relative ratio (RR) and 95% confidence interval (95% CI). Length of hospital stay is truncated at 100 days. **Table S3**. Association between congenital heart disease and length of hospital stay after delivery given by relative ratio (RR) and 95% confidence interval (95% CI). Length of hospital stay >50 days have been excluded. **Table S4**. Multilevel analysis of the association between congenital heart disease and length of hospital stay after delivery given by relative ratio (RR) and 95% confidence interval (95% CI).

## Data Availability

All data were provided by Statistics Denmark and due to their data privacy regulation, data with less than five individuals per cell were not reported. Data will not be made available to other researchers for the purpose of reproducing the results because this would be a violation of the Danish General Data protection Regulation [32] and data Privacy Regulation by Statistic Denmark [42].

## Abbreviations

CHD: Congenital Heart Disease
CI: Confidence Interval
DAG: Directed Acyclic Graph
ICD-8: International Classification of Diseases, 8th revision
ICD-10: International Classification of Diseases and Related Health Problems, 10th revision
ISCED: International Standard Classification of Education System
RR: Relative Ratio
