# Supplementary material for: Length of hospital stay after delivery among Danish women with congenital heart disease: a register-based cohort study
Source: BMC Pregnancy Childbirth. 2021 Dec 7;21:812. doi: 10.1186/s12884-021-04286-3 (PMC8650333; doi:10.1186/s12884-021-04286-3)
Supplement: Supplementary file 1 — Additional file 1 : Figure S1. Simplified Directed Acyclic Graph (DAG) highlighting variables of importance in the analysis of the association between maternal congenital heart disease and length of hospital stay. Table S1. Association between congenital heart disease and length of hospital stay after delivery given by relative ratio (RR) and 95% confidence interval (95% CI). Length of hospital stay is truncated at 30 days. Table S2. Association between congenital heart disease and length of hospital stay after delivery given by relative ratio (RR) and 95% confidence interval (95% CI). Length of hospital stay is truncated at 100 days. Table S3. Association between congenital heart disease and length of hospital stay after delivery given by relative ratio (RR) and 95% confidence interval (95% CI). Length of hospital stay >50 days have been excluded. Table S4. Multilevel analysis of the association between congenital heart disease and length of hospital stay after delivery given by relative ratio (RR) and 95% confidence interval (95% CI). [file 12884_2021_4286_MOESM1_ESM.docx]

**Supplementary material for:**

**Length of Hospital stay after delivery among Danish women with Congenital Heart Disease: A register-based cohort study**


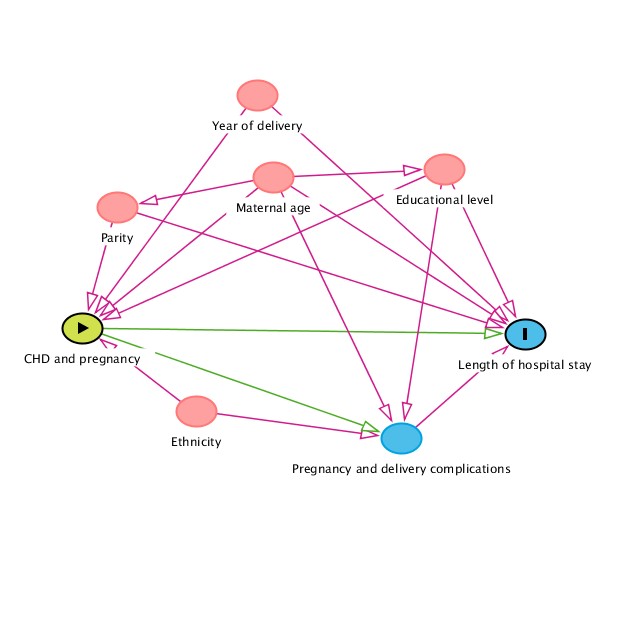

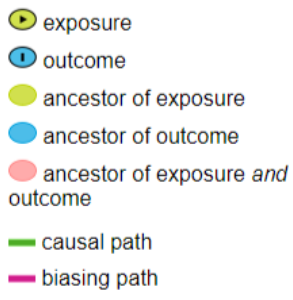


**Figure S1.** Simplified Directed Acyclic Graph (DAG) highlighting variables of importance in the analysis of the association between maternal congenital heart disease and length of hospital stay.

**Table S1.** Association between congenital heart disease and length of hospital stay after delivery given by relative ratio (RR) and 95% confidence interval (95% CI). Length of hospital stay is truncated at 30 days. Denmark, 1997-2014.

|  |  | Number of days of admission after delivery | | Unadjusted | | | Adjusted^#^ | | |
| --- | --- | --- | --- | --- | --- | --- | --- | --- | --- |
|  | N* | Mean (SD) | Median (IQR) | RR | 95 % CI | p-value | RR | 95 % CI | p-value |
| **Maternal congenital**  **heart disease** |  |  |  |  |  | <0.001 |  |  | <0.001 |
| No | 935,959 | 3.5 (3.3) | 3 (1-4) | 1 (ref) | - |  | 1 (ref) | - |  |
| Simple | 2,242 | 3.8 (3.7) | 3 (2-5) | 1.09 | 1.05-1.14 |  | 1.11 | 1.07-1.16 |  |
| Moderate | 1,093 | 4.0 (3.6) | 3 (2-5) | 1.14 | 1.07-1.21 |  | 1.15 | 1.08-1.22 |  |
| Complex | 384 | 4.8 (5.3) | 4 (2-5) | 1.37 | 1.20-1.56 |  | 1.39 | 1.22-1.59 |  |

*number of births in the unadjusted analysis

^#^adjusted for maternal age, year of delivery, parity, ethnicity, and educational level

**Table S2.** Association between congenital heart disease and length of hospital stay after delivery given by relative ratio (RR) and 95% confidence interval (95% CI). Length of hospital stay is truncated at 100 days. Denmark, 1997-2014.

|  |  | Number of days of admission after delivery | | Unadjusted | | | Adjusted^#^ | | |
| --- | --- | --- | --- | --- | --- | --- | --- | --- | --- |
|  | N* | Mean (SD) | Median (IQR) | RR | 95 % CI | p-value | RR | 95 % CI | p-value |
| **Maternal congenital**  **heart disease** |  |  |  |  |  | <0.001 |  |  | <0.001 |
| No | 935,959 | 3.6 (4.1) | 3 (1-4) | 1 (ref) | - |  | 1 (ref) | - |  |
| Simple | 2,242 | 4.0 (5.4) | 3 (2-5) | 1.12 | 1.05-1.19 |  | 1.14 | 1.07-1.21 |  |
| Moderate | 1,093 | 4.0 (4.2) | 3 (2-5) | 1.13 | 1.06-1.21 |  | 1.14 | 1.07-1.22 |  |
| Complex | 384 | 5.1 (6.8) | 4 (2-5) | 1.42 | 1.22-1.66 |  | 1.45 | 1.24-1.70 |  |

*number of births in the unadjusted analysis

^#^adjusted for maternal age, year of delivery, parity, ethnicity, and educational level

**Table S3.** Association between congenital heart disease and length of hospital stay after delivery given by relative ratio (RR) and 95% confidence interval (95% CI). Length of hospital stay >50 days have been excluded. Denmark, 1997-2014.

|  |  | Number of days of admission after delivery | | Unadjusted | | | Adjusted^#^ | | |
| --- | --- | --- | --- | --- | --- | --- | --- | --- | --- |
|  | N* | Mean (SD) | Median (IQR) | RR | 95 % CI | p-value | RR | 95 % CI | p-value |
| **Maternal congenital**  **heart disease** |  |  |  |  |  | <0.001 |  |  | <0.001 |
| No | 934,929 | 3.5 (3.4) | 3 (1-4) | 1 (ref) | - |  | 1 (ref) | - |  |
| Simple | 2,235 | 3.8 (3.6) | 3 (2-5) | 1.08 | 1.03-1.13 |  | 1.10 | 1.05-1.14 |  |
| Moderate | 1,092 | 4.0 (3.5) | 3 (2-5) | 1.13 | 1.07-1.20 |  | 1.15 | 1.08-1.21 |  |
| Complex | 383 | 4.9 (6.3) | 4 (2-5) | 1.41 | 1.21-1.64 |  | 1.44 | 1.23-1.68 |  |

*number of births in the unadjusted analysis

^#^adjusted for maternal age, year of delivery, parity, ethnicity, and educational level

**Table S4**. Multilevel analysis of the association between congenital heart disease and length of hospital stay after delivery given by relative ratio (RR) and 95% confidence interval (95% CI). Denmark, 1997-2014.

|  |  | Number of days of admission after delivery | | Unadjusted | | | Adjusted^#^ | | | |
| --- | --- | --- | --- | --- | --- | --- | --- | --- | --- | --- |
|  | N* | Mean (SD) | Median (IQR) | RR | 95 % CI | p-value | | RR | 95 % CI | p-value |
| **Maternal congenital**  **heart disease** |  |  |  |  |  | <0.001 | |  |  | <0.001 |
| No | 935,959 | 3.6 (3.7) | 3 (1-4) | 1 (ref) | - |  | | 1 (ref) | - |  |
| Simple | 2,242 | 3.9 (4.4) | 3 (2-5) | 1.10 | 1.06-1.14 |  | | 1.11 | 1.07-1.15 |  |
| Moderate | 1,093 | 4.0 (3.8) | 3 (2-5) | 1.14 | 1.08-1.19 |  | | 1.14 | 1.09-1.20 |  |
| Complex | 384 | 5.1 (6.7) | 4 (2-5) | 1.31 | 1.20-1.42 |  | | 1.32 | 1.22-1.43 |  |
|  |  |  |  |  |  |  | |  |  |  |
| Random effect (SE) |  |  |  | .50(0.00) | | | | 0.47(0.00) | | |

*number of births in the unadjusted analysis

^#^adjusted for maternal age, year of delivery, parity, ethnicity, and educational level
